# Associations Among Lifestyle Behaviors, Academic Achievement, and Physical Diseases in Adolescents: A Cross-Lagged Network Analysis

**DOI:** 10.3390/nu18030440

**Published:** 2026-01-29

**Authors:** Hui Xue, Chunyan Luo, Dongling Yang, Shuangxiao Qu, Yanting Yang, Xiaodong Sun, Wei Du, Fengyun Zhang

**Affiliations:** 1 School of Public Health, Southeast University, Nanjing 210009, China; 2 Department of Children and Adolescent Health, Shanghai Municipal Center for Disease Control and Prevention, Shanghai 201107, China; 3 Key Laboratory of Environmental Medicine Engineering, Ministry of Education, School of Public Health, Southeast University, Nanjing 210009, China

**Keywords:** adolescents, lifestyle behaviors, academic achievement, physical diseases, cross-lagged network analysis

## Abstract

**Objective:** We aimed to examine the longitudinal associations between lifestyle behaviors, academic achievement, and physical diseases in adolescents. **Study Design:** Longitudinal cohort study. **Methods:** We recruited participants (n = 4330; mean age of 14.0 (SD = 1.51) years at the first time point and 16.0 (1.51) years at the second time point) from 16 districts in Shanghai, China, who completed a survey in 2021 (T1) and 2023 (T2). We employed a cross-lagged panel network model to explore the interconnected relationships among lifestyle behaviors, academic achievement, and physical condition (i.e., obesity, high blood pressure, high myopia, depressive symptoms). **Results:** Among the cross-lagged associations, the predictive effects of T1 obesity on T2 high blood pressure (OR = 2.39), T1 breakfast skipping on T2 TV screen time (OR = 1.49), (in cross-domain relationships) T1 symptoms of depression on T2 low fruit and vegetable consumption (OR = 2.43), T1 obesity on T2 TV screen time (OR = 1.53), and T1 computer time on T2 high BP (OR = 1.31) were particularly prominent. Nonetheless, the observed cross-lagged effect sizes were small. Based on the sum of expected influence on their connecting nodes, obesity, depressive symptoms, and breakfast skipping demonstrated their paramount roles in the network metrics. We found breakfast skipping showed the strongest bridging effect among all factors in association with coexisting conditions and academic performance in children. **Conclusions:** Our findings identified breakfast skipping as the pivotal bridge node with the highest centrality within the network of modifiable lifestyle factors. Although this does not imply direct causality, its prominent bridge effect highlights its essential role in maintaining network stability and mediating interactions across distinct variable clusters.

## 1. Introduction

Childhood and adolescence represent a critical developmental period characterized by rapid physical growth, cognitive maturation, and behavioral habit formation [1]. Several key health-related lifestyle behaviors, including physical inactivity, excessive screen time, poor dietary habits, and breakfast skipping, often emerge or intensify during adolescence, establishing trajectories that are significantly associated with lifelong health and educational outcomes [2,3]. Concurrently, academic achievement serves as a pivotal indicator of cognitive and socioemotional development, influencing future social and economic success [4,5]. Alarmingly, the rising global prevalence of childhood physical diseases, such as obesity, myopia, high blood pressure, and mental disorders, poses a threat to immediate well-being and long-term health [6]. Recent data indicated that nearly 35% of adolescents worldwide exhibited at least three modifiable lifestyle risk factors for chronic diseases [7], while academic pressure-related complaints have surged in the past decade [8]. These interconnected challenges demand urgent investigation into their dynamic interrelationships.

Existing evidence suggests intricate, bidirectional associations among lifestyle behaviors, academic outcomes, and physical health. Prior studies indicated that engaging in regular physical activities could improve cognitive functions such as memory and executive control, which, in turn, would foster better academic achievement [9]. In contrast, extended exposure to electronic screens and unhealthy dietary behaviors were associated with elevated risks of obesity and cardiovascular complications in young people [10,11], which, in turn, led to reduced school engagement and poorer academic achievement [12]. High academic pressure and excessive study load often led to reduced physical activities; meanwhile, chronic physical conditions such as obesity were associated with increased school absence and diminished cognitive performance, indicating bidirectional relationships [12].

Psychological factors may mediate the aforementioned relationships. Academic stress was associated with unhealthy eating behaviors and increased screen time, while physical disease-associated depressive symptoms could affect academic motivation [13]. However, the majority of previous studies relied on a cross-sectional design that failed to capture temporal dynamics or causal pathways, often treating these factors as static rather than evolving processes [14,15]. Much attention has been paid to pairwise relationships (e.g., physical activities and academic achievements) rather than the complex network of interactions between multiple lifestyle behaviors, academic outcomes, and physical diseases.

In this study, we aimed to explore the dynamic connections between lifestyle behaviors, academic achievement, and physical diseases in young people using a cross-lagged network approach. Compared with the standard cross-lagged structural equation models, the cross-lagged panel network (CLPN) analysis was advantageous for its ability to simultaneously estimate complex interrelationships among multiple variables [16]. This approach can not only model the cross-lagged and autoregressive effects between variables but also intuitively present their complex associations through network visualization. In addition, by calculating centrality indicators, CLPN analysis enables the quantitative identification of important variables in the network matrices, thereby providing indications for potential intervention strategies. Given that network models do not prove causality even with cross-lagged data [17], this approach will identify temporal conditional associations among nodes within the network.

## 2. Methods

### 2.1. Study Design and Participants

We obtained data from the Surveillance for Common Diseases and Health Risk Factors among Students in Shanghai project, which was conducted from 2021 to 2023 and covered all 16 districts of Shanghai. A stratified, multi-stage cluster-sampling scheme was used for participant recruitment. In each district, five schools were randomly chosen, including two junior high schools, two senior high schools, and one vocational high school, covering all grades. A minimum of 80 students from every grade in each selected school were recruited. We established a linked cohort using anonymized personal identifiers to track individuals’ data annually from 2021 to 2023. In this study, Time 1 (T1) refers to the baseline data collection period (September–October 2021), and Time 2 (T2) refers to the follow-up assessment period conducted two years later (September–October 2023). We included 4330 participants who had complete annual data for lifestyle behaviors, physical diseases, and academic achievements over 3 years. While the question of representativeness could be avoided for the majority of cohort studies [18], the validity of extrapolating the current study sample to the general population would require a population-representing sample. In the absence of sufficient resources to achieve the ideal representativeness, we employed the aforementioned sample frame and selected 5 schools per district. In line with the national surveillance schemes, this pragmatic solution was reviewed and approved by the national expert committee [19]. Our study was reviewed and obtained approval from the Ethics Committee of the Shanghai Municipal Center for Disease Control and Prevention (Approval No. 2022-13).

### 2.2. Measures

#### 2.2.1. Physical Diseases

We considered four chronic diseases: high blood pressure (BP), high myopia, obesity, and symptoms of depression. More specifically, for adolescents aged 7 to 17 years, high BP is defined when systolic and/or diastolic values are above the 95th percentile corresponding to their sex, age, and height, where the thresholds are determined according to age-, sex-, and height-specific percentiles [20]. High myopia was defined as a spherical equivalent below −6.0 D based on results from screening myopia tests using non-cycloplegic autorefraction [21]. For participants aged 18 years and older, high BP was defined according to adult guidelines, with systolic blood pressure of 140 mmHg or higher and/or diastolic blood pressure of 90 mmHg or higher [20]. Obesity in adolescents under 18 years was defined as a body mass index (BMI) at or above the 95th percentile for their age and sex. For individuals aged 18 years and older, the adult criterion was used, with a BMI of 28.0 or higher indicating obesity [22]. Using adult cutoffs for obesity/BP at age 18 created artificial data discontinuities in a sample of adolescents (11–18 years). Allowing for the importance of benchmarking to achieve clinically meaningful improvement [23], we categorized BMI and BP readings into groups in line with the conventional good practice. Depressive symptoms were assessed using the 20-item Center for Epidemiological Studies Depression Scale. Participants indicated the frequency of each symptom over the previous week on a four-point scale ranging from 0 to 3. We categorized those with depressive symptoms as having greater than or equal to 20 points [24]. Dichotomization of variables based on well-established cutoff values to categorize, for example, outdoor activity time as sufficient or insufficient, computer time as <2 h/day or >=2 h/day, and depressive symptoms as present or absent, would provide actionable definitions of population groups for targeted public health surveillance and intervention programs.

#### 2.2.2. Lifestyle Behaviors

Lifestyle variables were collected using the conventional Students’ Health-Risk Behaviors Surveillance Questionnaire, which was developed based on the U.S. Youth Risk Behavior Survey (YRBS) and the Chinese Adolescent Health-Risk Behavior framework [19]. Validation of this instrument has been carried out during the pilot stage in China and reported elsewhere [25]; e.g., the median values of test–retest reliability range from 89.3% to 92.3% for various dietary behaviors and 90.5% to 95.7% for the other lifestyle factors among high school children and adolescents.

##### Low Consumption of Fruits and Vegetables

Fruit and vegetable consumption was evaluated by asking participants how many times per day they usually consumed fruits and vegetables over the previous seven days. Low consumption was defined as eating fruits or vegetables less than one time per day.

##### Consumption of Sugar-Sweetened Beverages

Sugar-sweetened-beverage consumption was assessed by asking participants how often they consumed such drinks each day during the previous seven days. Consumption was classified as yes or no according to whether participants reported daily consumption in the past week.

##### Breakfast Skipping

Breakfast habits were assessed by asking participants whether they ate breakfast every day in the previous week. Individuals who reported daily breakfast consumption were classified as breakfast eaters, while those who did not were considered to have a habit of skipping breakfast.

##### Outdoor Activity Time

Outdoor activity time was categorized as ‘yes’ or ‘no’ based on whether individuals engaged in at least 2 h of outdoor activities per day in the past week. Those who met this criterion were classified as ‘yes’, while those who did not were classified as ‘no’ [26].

##### TV Screen Time

TV screen time was classified as less than 2 h per day or 2 h or more per day, according to participants’ responses to the question regarding their average daily TV viewing in the previous week.

##### Computer Time

Computer time was classified as less than 2 h per day or 2 h or more per day, according to participants’ responses about their average daily computer use in the previous week.

##### Screen Time on Mobile Devices

Screen time on mobile devices was calculated and classified as either less than 2 h per day, indicating no prolonged screen use, or 2 h per day or more, indicating prolonged screen use.

#### 2.2.3. Academic Achievement

Participants self-reported their academic achievements by answering the question “Compared with your classmates, how would you rate your academic achievement”. Participants responded with one of the options, ‘excellent’, ‘above average’, ‘average’, ‘below average’, or ‘poor’. For analysis, these responses were categorized into two groups, with ‘excellent’ as one group and all the other responses merged into the ‘general’ group.

#### 2.2.4. Covariates

All participants provided anonymous self-reported responses to the structured questionnaire administered in a classroom setting under the guidance of trained staff. Responses included their date of birth (to be subtracted from the autogenerated survey date and then divided by 365.25 to convert into age in years), sex (i.e., boy or girl), type of school (i.e., junior high school, senior high school, or vocational school), and maternal and paternal education attainment (i.e., primary school or below, junior high, senior high, vocational school, college or above) at T1.

### 2.3. Statistical Analysis

All analyses in this study were performed using R language (Version 4.4.2; R Foundation for Statistical Computing, Vienna, Austria). We summarized categorical variables as frequencies and percentages and presented continuous variables as means and standard deviations. We used the Shapiro–Wilks test to evaluate the normality of continuous quantitative variables. Since all continuous variables were not normally distributed, we transformed them into dichotomous categories to enhance interpretability. We compared the baseline characteristics of participants included in the analysis with those who were excluded due to incomplete annual data. Differences between the included and excluded participants were assessed using independent t-tests for continuous variables and Chi-square tests for categorical variables. We applied the CLPN approach to the overall sample and then conducted sex-specific subgroup analyses. To establish the CLPN model, L1-regularized logistic regression was initially employed to obtain the autoregressive as well as cross-lagged coefficients, which allowed the calculation of lagged associations across the two time points. The autoregressive coefficients reflected the extent to which a variable at T1 would predict its own value at T2, after controlling for other T1 variables. The cross-lagged coefficients indicated how a T1 variable would predict different T2 outcomes, with adjustment for all other variables at T1. Relative risk estimation is one of the common practices for cohort studies. However, in the presence of complicated network matrices, leading to substantial computation time for high-dimensional nodewise estimation of relative risks, we opted to use the nodewise L1-regularized logistic regression to improve model efficiency and stability, as well as the extended Bayesian information criterion for model selection to quantify nodewise relationships and establish the edge weights for the network [27]. We employed the glmnet package to conduct regularized regressions and the qgraph package to generate visualizations of network graphs. Specifically, thicker edges would indicate stronger associations. The logistic regression coefficients representing edge weights were transformed from log odds to odds ratios (ORs). High OR-based weights reflect the strength of local associations, and do not automatically imply strong predictive influence in networks.

Although network visualization can display the relevance between nodes and their interconnections in an intuitive way, interpretation based solely on the spatial layout of nodes was not precise enough to fully reflect the modeling results [28]. To obtain a more detailed understanding of the network, we derived several quantitative indicators. For each node, we calculated two centrality metrics, including cross-lagged out-expected influence (out-EI) and in-expected influence (in-EI). Out-EI represents the predictive power of T1 nodes over T2 nodes, while in-EI reflects the predictive power of T2 nodes relative to T1 nodes. Notably, these metrics excluded autoregressive paths. In addition, we examined potential bridge nodes by estimating bridge EI with the bridge function in the networktools package. This index indicates the degree to which a node is connected to the other nodes belonging to different domains [29]. Centrality indicators are presented in standardized form using Z scores.

The precision of edge weight estimates and the stability of centrality indicators were assessed using the bootnet package [30]. A nonparametric bootstrap procedure with 1000 repetitions was performed to calculate 95-percent confidence intervals for all edge weights. The width of the confidence intervals provides a direct indication of the precision of the estimates, with narrower intervals reflecting greater accuracy. To assess the stability of centrality measures, 1000 case-deletion bootstrap resamples were performed to estimate the correlation stability (CS) coefficient. A CS coefficient exceeding 0.25 is generally regarded as indicating acceptable stability, while values above 0.50 are considered to reflect high stability [30].

To explore the structural differences between boys’ and girls’ networks, we adhered to the standardized network comparison procedure within the CLPN framework [31]. Specifically, at the edge level, the global similarity of the two networks was quantified by calculating the correlation between their edge weight matrices. Furthermore, the proportion of edges with consistent directions across sex groups was computed to assess structural consistency. At the node level, we evaluated the consistency of centrality indicators to identify nodes that ranked prominently in both sex groups, thereby elucidating the functional stability of nodes between different sex groups.

## 3. Results

We included 4330 adolescents (53.9% were boys), with a mean age of 14.0 (SD = 1.51) years at the first time point and 16.0 (1.51) years at the second time point. Table 1 presents the counts and proportions of items related to lifestyle behaviors, academic achievements, and physical diseases, as assessed at both baseline and follow-up periods, stratified by sex (Table 1). Significant differences from baseline were observed between the included (n = 4330) and excluded (n = 10,727) participants across several variables, including age (inclusion 14.0 years; exclusion 15.0 years), breakfast skipping (14.8%; 18.8%), outdoor activity time (28.2%; 26.5%), computer time (9.5%; 12.8%), mobile screen time (39.3%; 30.7%), high BP (16.4%; 19.0%), high myopia (8.1%; 11.0%), and depressive symptoms (14.8%; 19.2%) (Table S1).

**Figure 1:**
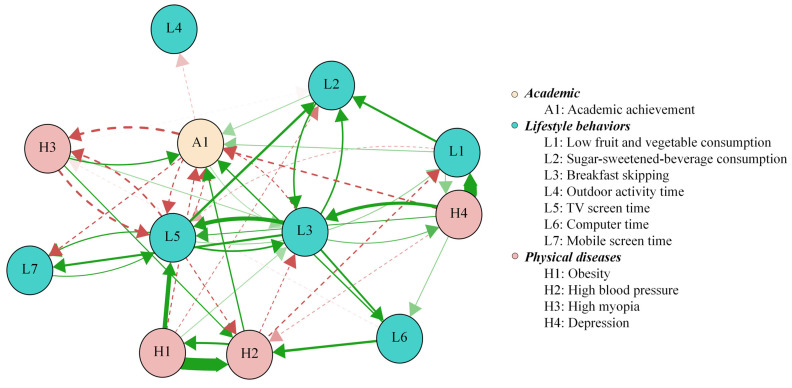
Cross-lagged panel network of associations among lifestyle behaviors, academic achievements, and physical diseases in total sample. Note: The arrows represent the unidirectional predictive effect of one node at T1 on another node at T2. The colors represent the direction of the association: green solid edges denote positive associations (i.e., odds ratios > 1), and red dashed edges denote negative associations (i.e., odds ratios < 1). Edge thickness represents the strength of the odds ratio, with thicker edges corresponding to stronger cross-lagged effects. Autoregressive edges and covariates were excluded from the plot to enhance visual clarity.

**Figure 2:**
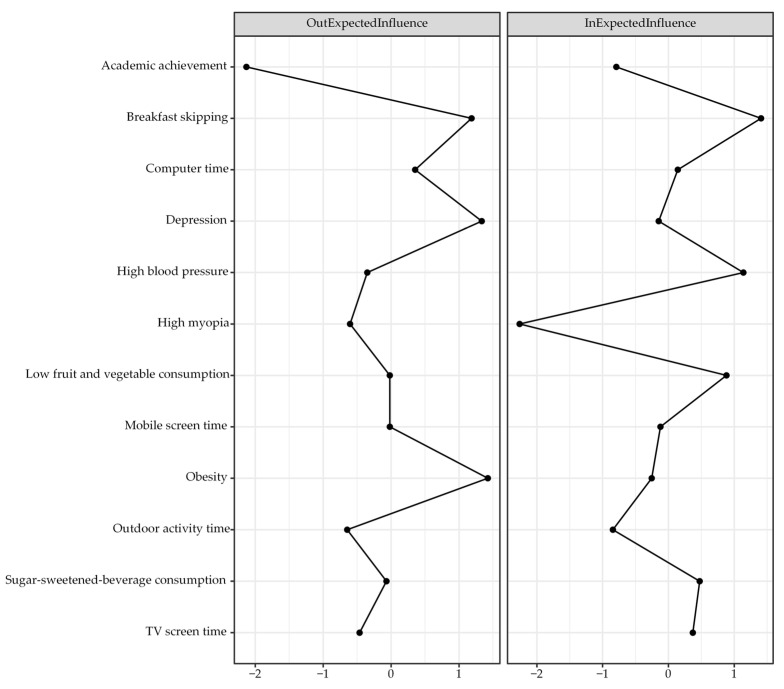
Out-expected influence and in-expected influence centrality estimates in the CLPN. Larger values reflect greater centrality.

**Figure 3:**
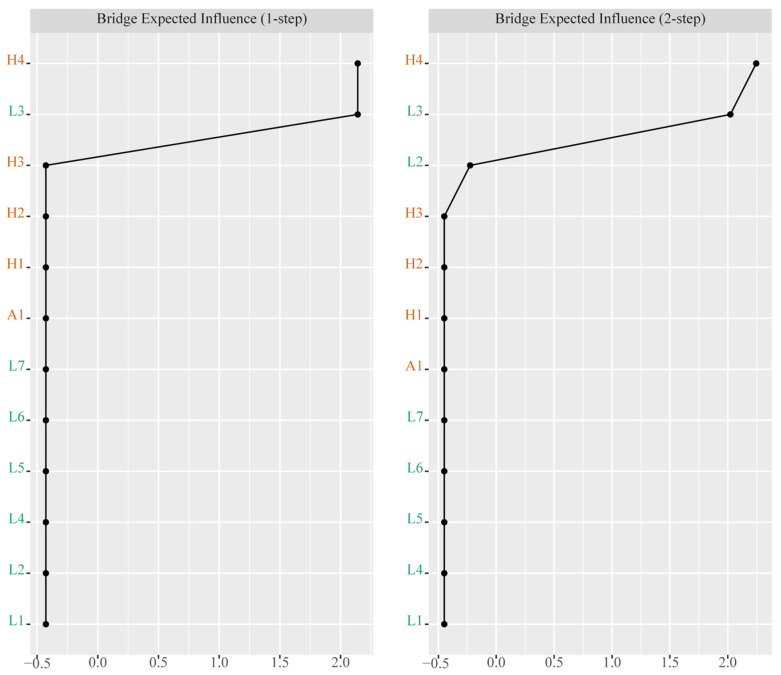
Two-step bridge expected influence for contemporaneous networks. Note. Labels were color-coded to indicate variable categories: orange labels denoted physical diseases and academic achievement, while green labels denoted lifestyle behaviors.

Figure 1 presents the CLPN with only cross-lagged pathways in the total sample. The arrows indicate pairwise cross-lagged relations among nodes while adjusting for all the other variables at T1. Edge weights across the total sample are reported in Table 2. Autoregressive effects were statistically greater than cross-lagged effects, with mean odds ratios (OR) of 31.46 and 1.05, respectively. The observed cross-lagged effects were rather negligible, especially when compared to the substantially larger autoregressive effects, indicating state shifting over time for these constructs. The three largest cross-lagged effects within the same domain were observed from obesity at T1 to high BP at T2, with an OR of 2.39, and from breakfast skipping at T1 to TV screen time at T2, with an OR of 1.49. In addition, three relatively strong cross-lagged associations involving different domains were identified. Depressive symptoms at T1 predicted lower fruit and vegetable consumption at T2, with an OR of 2.43. Obesity at T1 was associated with TV screen time at T2, with an OR of 1.53. Computer use at T1 was associated with high BP at T2, with an OR of 1.31. We used the cross-lagged analysis to describe reciprocal relationships between variables from one to another and vice versa, as well as across different time points. While these cross-lagged connections were theoretically meaningful, they should be interpreted within the context of robust temporal stability. Figure 2 presents the centrality values. Obesity, depressive symptoms, and breakfast skipping show higher out-EI than the other nodes, suggesting stronger connections with the subsequent nodes. Breakfast skipping, high BP, and low fruit and vegetable consumption show higher in-EI than the other nodes, implying that they were more likely to be affected by preceding variables. Notably, the nodes with the two highest bridge EIs were depressive symptoms and breakfast skipping (Figure 3).

**Figure 4:**
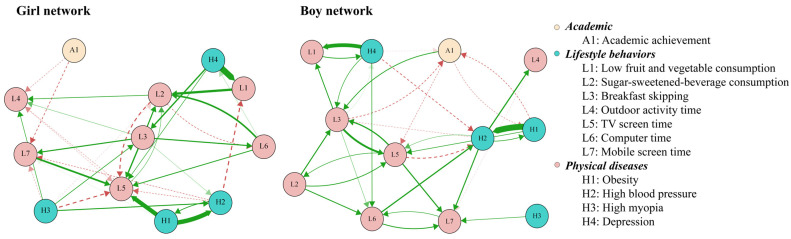
Cross-lagged panel networks in boys and girls. Note: The arrows represent the unidirectional predictive effect of one node at T1 on another node at T2. The colors represent the direction of the association: green solid edges denote positive associations (i.e., odds ratios > 1), and red dashed edges denote negative associations (i.e., odds ratios < 1). Edge thickness represents the strength of the odds ratio, with thicker edges corresponding to stronger cross-lagged effects. Autoregressive edges and covariates were excluded from the plot to enhance visual clarity.

**Figure 5:**
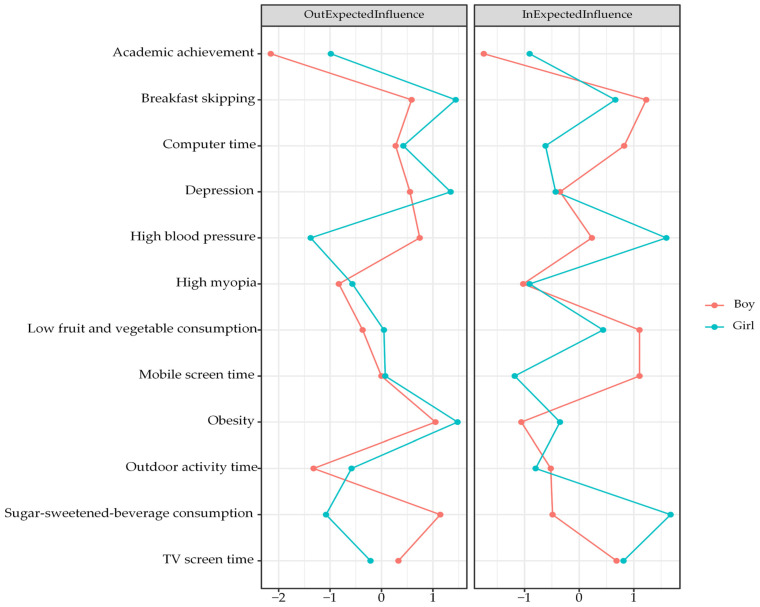
Out-expected influence and in-expected influence centrality estimates in boys and girls.

Supplementary Table S1 reports the edge weights for boys. Two relatively strong cross-lagged associations within the same construct were observed from T1 obesity to T2 high BP (OR = 2.13) and from T1 skipping breakfast to T2 TV screen time (OR = 1.40). In addition, three relatively strong cross-lagged associations across different domains were identified. These included the paths from T1 depression to T2 low fruit and vegetable consumption (OR = 1.74), from T1 computer time to T2 high BP (OR = 1.29), and from T1 high BP to T2 outdoor activity time (OR = 1.28) (Figure 4). Centrality estimates for boys (Figure 5) indicated that sugar-sweetened-beverage consumption had higher out-EI than other nodes, implying it consistently led to subsequent changes in the network. Breakfast skipping displayed higher in-EI, indicating that it was likely to follow after the presence of other nodes.

Supplementary Table S3 presents the edge weights for girls. Two strong within-domain cross-lagged connections were identified from T1 obesity to T2 high BP (OR = 2.58) and from T1 low fruit and vegetable consumption to T2 sugar-sweetened-beverage consumption (OR = 1.82). In addition, three relatively strong cross-lagged connections across different domains were observed, i.e., from T1 depressive symptoms to T2 low fruit and vegetable consumption (OR = 3.12), from T1 obesity to T2 TV screen time (OR = 2.50), and from T1 depressive symptoms to T2 breakfast skipping (OR = 1.47) (Figure 4). In addition, the centrality estimates for girls (Figure 5) showed that obesity displayed higher out-EI than the other nodes, implying a stronger effect on subsequent nodes in the network. In addition, sugar-sweetened-beverage consumption showed higher in-EI than the other nodes, implying the likelihood to occur after the presence of other factors.

The structural differences between boys’ and girls’ networks revealed a moderate, statistically significant global similarity in edge weights (r = 0.45, *p* < 0.001, 95% CI: 0.30, 0.57), with a Jaccard index of 0.36 indicating that 36% of edges were shared with the same sign across groups. Specifically, 49.3% of the edges in the boys’ network and 50.7% in the girls’ network were replicated in the other, while the centrality profiles of the two networks showed no significant correlation of either out-EI (r = 0.39, *p* = 0.212) or in-EI (r = 0.28, *p* = 0.384). Further assessment of rank consistency in node centrality showed that no nodes had matching rank-order for out-EI between sex groups, while only three nodes (outdoor activity time, high myopia, and depression) exhibited consistent rank alignment for in-EI.

Results from the edge-weight bootstrap analyses (Supplementary Figures S1 and S2) indicate that the networks demonstrated a moderate level of accuracy. For the total sample, the CS coefficients are 0.52 for edges, 0.36 for in-EI, and 0.52 for out-EI. Among boys, the corresponding values are 0.21, 0.28, and 0.28, and among girls, they are 0.36, 0.28, and 0.52. These values indicate that the overall network and the sex-specific networks show acceptable stability (Supplementary Figures S3 and S4). The results of the edge weight difference and the centrality difference are shown in Supplementary Figures S5–S9. Notably, the strongest edges and items with the highest in-EI and out-EI are significantly different from their weakest and lowest counterparts.

## 4. Discussion

Using three-year longitudinal linked cohort data from the Shanghai region of China, our study revealed the complex connections between behaviors and health status among adolescents. Previous studies mostly focused on unidirectional associations between single or multiple behaviors and specific health indicators [14,15,32,33]. In contrast, our study further demonstrated that such associations were actually dynamically intertwined, which not only manifested as the potential impact of behaviors on physical health (e.g., the associations between computer use time and high BP) but also demonstrated the likely reversed influence of health status on behavioral choices (e.g., the association between obesity and subsequent TV screen time). Above all, we found that breakfast skipping serves as a key bridging node among modifiable behavioral factors, playing a central connecting role in the associations between indicators in the behavioral and health domains.

This cross-lagged network analysis of twelve nodes identified breakfast skipping as having strong outward and inward edges, indicating its prominent role in the network over time. While breakfast skipping acts as an important receiver of cross-lagged effects from other variables, it, along with other lifestyle factors, exerts robust cross-lagged associations with multiple interconnected nodes. Moreover, breakfast skipping also functions as a bridging phenomenon connecting lifestyle behaviors and physical conditions, which aligns with prior cross-sectional studies documenting associations between breakfast skipping, unhealthy lifestyle patterns, elevated risk of physical morbidities, and suboptimal academic outcomes among adolescents [11,15,34,35]. The current findings highlight the differential structural positions of lifestyle patterns in shaping adolescent academic achievement and physical conditions, without implying causal directionalities. Future studies may validate these network configurations across diverse adolescent cohorts.

Out of all behavioral factors, breakfast skipping has the strongest association with TV screen time. Previous studies found that regular breakfast habits were one of the core indicators of structured daily routines for children, and the observed association seemed to reflect a loss of control over allocation of time [36]. Skipping breakfast in children may lead to low blood sugar and insufficient energy, thereby making them tend to choose activities with low physical exertion [37]. Consistent with the current finding, one study on 177,091 Greek school children showed that those who skipped breakfast were more likely to have excessive screen time [35]. Intervention strategies might consider addressing the development of regular breakfast consumption habits, thereby facilitating a shift from low-energy, screen-based behaviors to higher-intensity physical activities among children.

Among various physical diseases examined in this study, obesity exhibits the most significant association with high BP. Previous research indicated that approximately 60–70% of obese children suffered from insulin resistance, and the resultant elevated insulin levels could then activate excessive sympathetic nervous system activities, thereby leading to increased heart rate and vasoconstriction [38]. In addition, adipose tissue in obese children might release large amounts of pro-inflammatory cytokines and free fatty acids, which could induce chronic low-grade inflammation and oxidative stress responses [39]. One obvious speculation exists, in that these processes would potentially impair the vascular endothelial integrity, resulting in reduced vascular dilation function and increased stiffness. Furthermore, the previous study demonstrated that obese children face a 3-fold higher risk of hypertension compared to their normal-weight peers [40], which was consistent with the current finding. As a critical period for cardiovascular system development, attentions should be paid to cardiovascular health during childhood, allowing for that obesity-induced elevated blood pressure often emerged during school age and potentially progressed to persistent hypertension with the sustained presence of obesity. Aligned with China’s policy orientation of co-prevention of multiple diseases, the current study provided additional evidence on the longitudinal connections between multiple diseases, in response to the current global commitment to safeguarding the health of adolescents.

Our study has several limitations. First, the current study employed self-reports in the network analysis, potentially leading to recall or social desirability biases for potential explanatory pathways. The lack of empirical examination of physiological mechanisms also restricted our ability to identify cause-effect associations. Given the observational nature of the study, causal inference cannot be established. Considering the nature of network models as an exploratory but data-driven approach, the current findings could inform future hypotheses in regards with potential causal relationships. Second, the dichotomization of continuous variables inevitably led to a loss of information and reduced statistical power, and therefore low stability results should be interpreted with caution. Nonetheless, the alternative approach using continuous variables revealed little material changes to the current primary findings. Hence the current findings were somewhat robust with great interpretability for public health policy and practice. Third, the lack of empirical examination of physiological mechanisms also restricted our ability to identify cause-effect associations. In addition, we need to be cautious when interpreting OR-based edges from LASSO-regularization, which could introduce estimation bias especially in finite samples. Rather than as precise effect sizes, these ORs should be interpreted as indicators of the direction and relative importance of associations. Furthermore, the potential for attrition bias in the complete data analytic framework would warrant a cautious interpretation of the current findings. Given that linked cohort studies inherently require longitudinal data completeness, the exclusion of individuals with missing data reflected the lack of generalizabilty in the current setting.

Of a range of modifiable lifestyle factors, breakfast skipping demonstrated the strongest bridging effect, as a pivotal bridge node, exhibiting high bridge centrality within the network structure. Rather than implying direct causality, the identification of breakfast skipping’s bridging role highlighted the dynamic interplay and longitudinal predictability inherent in the network. Considering the central role of breakfast skipping in maintaining network stability in the current setting, future confirmatory efforts may invest on its potential to facilitate the transmission of influence across distinct clusters of variables over time.

## 5. Conclusions

Our findings demonstrated the complex interplay among lifestyle factors, physical conditions, and academic achievements in adolescents, and highlighted the importance to consider the network structure and components for an integrated multimodal lifestyle intervention strategy. Future efforts are warranted to investigate the potential underlying mechanisms to inform effective lifestyle solutions for better health and development in adolescents.

## Figures and Tables

**Table 1:** Descriptive statistics of lifestyle behaviors, academic achievements, and physical diseases among adolescents in the cross-lagged panel network across two time points.

Items	Label	Total Sample (n = 4330)		Boys (n = 2232)		Girls (n = 2098)	
		Time 1	Time 2	Time 1	Time 2	Time 1	Time 2
Age, year (Mean, SD)		14.0 (1.51)	16.0 (1.51)	14.1 (1.52)	16.1 (1.52)	14.0 (1.50)	16.0 (1.49)
Lifestyle behaviors							
Low fruit and vegetable consumption	L1						
No		4171 (96.3)	4097 (94.6)	2133 (95.6)	2082 (93.3)	2038 (97.1)	2015 (96.0)
Yes		159 (3.7)	233 (5.4)	99 (4.4)	150 (6.7)	60 (2.9)	83 (4.0)
Sugar-sweetened-beverage consumption	L2						
No		3734 (86.2)	3654 (84.4)	1875 (84.0)	1825 (81.8)	1859 (88.6)	1829 (87.2)
Yes		596 (13.8)	676 (15.6)	357 (16.0)	407 (18.2)	239 (11.4)	269 (12.8)
Breakfast skipping	L3						
No		3689 (85.2)	3469 (80.1)	1953 (87.5)	1816 (81.4)	1736 (82.7)	1653 (78.8)
Yes		641 (14.8)	861 (19.9)	279 (12.5)	416 (18.6)	362 (17.3)	445 (21.2)
Outdoor activity time	L4						
Insufficient		3109 (71.8)	3367 (77.8)	1553 (69.6)	1693 (75.9)	1556 (74.2)	1674 (79.8)
Sufficient		1221 (28.2)	963 (22.2)	679 (30.4)	539 (24.1)	542 (25.8)	424 (20.2)
TV screen time	L5						
<2 h/day		3839 (88.7)	3893 (89.9)	1936 (86.7)	1962 (87.9)	1903 (90.7)	1931 (92.0)
>=2 h/day		491 (11.3)	437 (10.1)	296 (13.3)	270 (12.1)	195 (9.3)	167 (8.0)
Computer time	L6						
<2 h/day		3920 (90.5)	3712 (85.7)	1983 (88.8)	1803 (80.8)	1937 (92.3)	1909 (91.0)
>=2 h/day		410 (9.5)	618 (14.3)	249 (11.2)	429 (19.2)	161 (7.7)	189 (9.0)
Mobile screen time	L7						
<2 h/day		3000 (69.3)	2582 (59.6)	1547 (69.3)	1367 (61.2)	1453 (69.3)	1215 (57.9)
>=2 h/day		1330 (30.7)	1748 (40.4)	685 (30.7)	865 (38.8)	645 (30.7)	883 (42.1)
Academic							
Academic achievement	A1						
General		3930 (90.8)	3777 (87.2)	1987 (89.0)	1896 (84.9)	1943 (92.6)	1881 (89.7)
Excellent		400 (9.2)	553 (12.8)	245 (11.0)	336 (15.1)	155 (7.4)	217 (10.3)
Physical diseases							
Obesity	H1	761 (17.6)	743 (17.2)	498 (22.3)	513 (23.0)	263 (12.5)	230 (11.0)
High blood pressure	H2	708 (16.4)	847 (19.6)	371 (16.6)	484 (21.7)	337 (16.1)	363 (17.3)
High myopia	H3	352 (8.1)	477 (11.0)	179 (8.0)	259 (11.6)	173 (8.2)	218 (10.4)
Symptoms of depression	H4	641 (14.8)	911 (21.0)	265(11.9)	464 (20.8)	376 (17.9)	447 (21.31)

**Table 2:** Odds ratio for each cross-lagged network association of the total sample.

	L1	L2	L3	L4	L5	L6	L7	A1	H1	H2	H3	H4
L1	4.60	1.28	1.00	1.00	0.88	1.00	1.00	1.13	1.00	1.00	1.00	1.13
L2	1.00	2.71	1.22	1.07	1.00	1.00	1.00	1.12	1.00	0.94	1.00	1.00
L3	1.00	1.23	3.66	1.00	1.49	1.26	1.28	0.84	1.00	1.00	1.00	1.14
L4	1.00	1.00	1.00	1.67	1.00	1.00	0.97	1.03	1.00	1.00	1.00	1.00
L5	1.13	1.31	1.24	0.98	1.77	1.00	1.18	0.84	1.00	0.82	0.76	1.00
L6	1.00	1.05	1.02	1.00	1.05	2.48	1.06	1.20	1.00	1.31	0.89	1.05
L7	1.00	1.05	1.10	1.00	1.15	1.09	2.40	0.99	1.00	1.00	1.00	1.00
A1	1.00	0.95	1.12	0.88	0.81	1.00	0.82	7.54	1.00	1.00	0.69	1.00
H1	1.00	0.86	1.12	1.00	1.53	1.00	1.07	0.82	58.64	2.39	1.04	1.00
H2	0.81	1.00	0.85	1.08	1.05	1.00	1.00	1.20	1.27	4.42	1.00	0.99
H3	0.99	0.90	1.12	1.00	0.73	1.00	1.00	1.20	1.00	1.17	284.36	1.00
H4	2.43	1.09	1.39	0.96	1.15	1.13	1.00	0.77	1.00	0.87	1.00	3.29

Note. Adjacency matrix of the T1-to-T2 cross-lagged panel network in the total sample. Independent variables (i.e., predictors) are in columns, and dependent variables are in rows. Autoregressive edges are presented along the diagonal. L1 = low fruit and vegetable consumption; L2 = sugar-sweetened-beverage consumption; L3 = breakfast skipping; L4 = outdoor activity time; L5 = TV screen time; L6 = computer time; L7 = mobile screen time; A1 = academic achievement; H1 = obesity; H2 = high blood pressure; H3 = high myopia; H4 = depression.

## Data Availability

All relevant data are shown within the manuscript, but original datasets cannot be shared because of involving students’ personal privacy.

## Supplementary Materials

The following supporting information can be downloaded at: https://www.mdpi.com/article/10.3390/nu18030440/s1. Table S1. Comparison of baseline characteristics between students with complete annual data (included) and those without (excluded); Table S2. Odds ratio for each cross-lagged network association in boys; Table S3. Odds ratio for each cross-lagged network association in girls; Figure S1. Edge weight accuracy for the network of the total sample; Figure S2. Edge weight accuracy for networks by sex; Figure S3. Stability of the centrality measures for the network of the total sample; Figure S4. Stability of the centrality measures for networks by sex; Figure S5. Edge weight difference tests in the network of the total sample; Figure S6. Edge weight difference tests for the networks by sex; Figure S7. Out-EI and in-EI centrality difference tests in the total sample; Figure S8. Out-EI and in-EI centrality difference tests in boys; Figure S9. Out-EI and in-EI centrality difference tests in girls.
