# Supplementary material for: Associations Among Lifestyle Behaviors, Academic Achievement, and Physical Diseases in Adolescents: A Cross-Lagged Network Analysis
Source: Nutrients. 2026 Jan 29;18(3):440. doi: 10.3390/nu18030440 (PMC12899500; doi:10.3390/nu18030440)

Associations among lifestyle behaviors, academic achievement, and physical diseases in adolescents: A cross-lagged network analysis

Supplementary information includes:

Table S1. Comparison of baseline characteristics between students with complete annual data (included) and those without (excluded).

Table S2. Odds ratio for each cross-lagged network association in boys.

Table S3. Odds ratio for each cross-lagged network association in girls.

Figure S1. Edge weight accuracy for the network of the total sample.

Figure S2. Edge weight accuracy for networks by sex.

Figure S3. Stability of the centrality measures for the network of the total sample.

Figure S4. Stability of the centrality measures for networks by sex.

Figure S5. Edge weight difference tests in the network of the total sample.

Figure S6. Edge weight difference tests for the networks by sex.

Figure S7. Out-EI and in-EI centrality difference tests in the total sample.

Figure S8. Out-EI and in-EI centrality difference tests in boys.

Figure S9. Out-EI and in-EI centrality difference tests in girls.

Table S1. Comparison of baseline characteristics between students with complete annual data (included) and those without (excluded).

Items	Total students surveyed in 2021 (n=15056)		*P*-value
	Included (n=4330)	Excluded (n=10727)	
Age, year (Mean, SD)	14.0 (1.51)	15.0 (1.74)	<0.001
Lifestyle behaviors			
Low fruit and vegetable consumption			0.115
No	4170 (96.3)	10271 (95.7)	
Yes	159 (3.7)	456 (4.3)	
Sugar-sweetened-beverage consumption			1.000
No	3733 (86.2)	9251 (86.2)	
Yes	596 (13.8)	1476 (13.8)	
Breakfast skipping			<0.001
No	3689 (85.2)	8714 (81.2)	
Yes	640 (14.8)	2013 (18.8)	
Outdoor activity time			0.036
Insufficient	3108 (71.8)	7877 (73.5)	
Sufficient	1221 (28.2)	2842 (26.5)	
TV screen time			0.346
< 2 h/day	3839 (88.7)	9450 (88.1)	
>= 2 h/day	491 (11.3)	1277 (11.9)	
Computer time			<0.001
< 2 h/day	3919 (90.5)	9355 (87.2)	
>= 2 h/day	410 (9.5)	1372 (12.8)	
Mobile Screen time			<0.001
< 2 h/day	6504 (60.7)	3000 (69.3)	
>= 2 h/day	4217 (39.3)	1330 (30.7)	
Academic			
Academic achievement			0.098
General	3930 (90.8)	9637 (89.9)	
Excellent	400 (9.2)	1088 (10.1)	
Physical diseases			
Obesity	761 (17.6)	1693 (15.8)	0.007
High blood pressure	708 (16.4)	2036(19.0)	<0.001
High myopia	352 (8.1)	1178 (11.0)	<0.001
Depressive symptoms	641(14.8)	2063 (19.2)	<0.001

Table S2. Odds ratio for each cross-lagged network association in boys.

	L1	L2	L3	L4	L5	L6	L7	A1	H1	H2	H3	H4
L1	3.81	1.00	1.04	1.00	1.00	1.00	1.00	1.02	1.00	1.00	1.00	1.20
L2	1.00	2.40	1.25	1.00	1.21	1.23	1.08	1.04	1.00	1.00	1.00	1.00
L3	1.23	1.00	3.21	1.00	1.40	1.13	1.08	0.83	1.00	0.90	1.00	1.11
L4	1.00	1.00	1.00	1.53	1.00	1.00	0.89	1.08	0.97	1.00	1.00	1.00
L5	1.00	1.17	1.29	1.00	1.65	1.08	1.27	1.00	1.00	0.78	1.00	1.00
L6	1.00	1.00	1.00	1.00	1.00	2.23	1.22	1.00	1.00	1.29	1.00	1.00
L7	1.00	1.07	1.09	1.00	1.00	1.16	2.43	1.07	1.00	1.00	1.00	1.00
A1	1.00	1.00	1.21	0.93	0.86	0.94	0.91	5.88	0.86	1.00	1.00	1.00
H1	1.00	0.97	1.00	1.00	1.18	1.00	1.00	0.84	52.07	2.13	1.00	1.00
H2	1.00	1.00	0.87	1.28	1.14	1.00	1.26	1.02	1.18	4.41	1.00	0.95
H3	1.00	1.00	1.00	1.00	0.94	1.00	1.16	1.00	1.00	1.00	154.24	1.00
H4	1.74	1.00	1.17	1.00	1.00	1.18	1.00	0.89	1.00	0.81	1.00	3.26

Note. Adjacency matrix of the T1 to T2 cross-lagged panel network in boys. Independent variables (i.e., predictors) are in columns, and dependent variables are in rows. Autoregressive edges are presented along the diagonal.

L1 = low fruit and vegetable consumption; L2 = sugar-sweetened-beverage consumption; L3 = breakfast skipping; L4 = outdoor activity time; L5 = TV screen time; L6 = computer time; L7 = mobile screen time; A1 = academic achievement; H1 = obesity; H2 = high blood pressure; H3 = high myopia; H4=depression.

Table S3. Odds ratio for each cross-lagged network association in girls.

	L1	L2	L3	L4	L5	L6	L7	A1	H1	H2	H3	H4
L1	5.07	1.82	1.00	1.00	0.91	1.00	1.00	1.00	1.00	1.00	1.00	1.00
L2	1.00	2.88	1.05	1.21	0.63	0.82	1.00	1.00	1.00	0.91	1.00	1.00
L3	1.00	1.41	3.84	1.13	1.47	1.38	1.38	1.00	1.05	1.12	1.00	1.10
L4	1.00	1.07	1.00	1.72	0.87	1.00	1.00	1.00	1.00	1.00	1.00	1.00
L5	1.05	1.14	1.05	1.00	1.70	1.00	1.00	1.00	1.00	1.00	1.00	1.03
L6	1.00	1.56	1.00	1.01	1.30	2.82	1.00	1.00	1.00	1.01	1.00	1.12
L7	1.00	1.00	1.05	1.00	1.60	1.00	2.26	1.00	1.00	1.00	1.00	1.00
A1	1.00	1.00	1.00	0.86	0.97	1.00	0.78	8.61	1.00	1.00	1.00	1.00
H1	1.00	0.91	1.06	0.87	2.50	1.00	1.11	1.00	67.45	2.58	1.00	1.00
H2	0.60	0.91	1.00	0.92	0.85	1.00	0.84	1.00	1.26	4.08	1.00	1.00
H3	1.00	0.89	1.22	1.19	0.63	1.00	0.87	1.00	1.00	1.34	236.69	1.00
H4	3.12	1.03	1.47	0.92	1.18	1.03	1.00	1.00	1.00	1.00	1.00	3.19

Note. Adjacency matrix of the T1 to T2 cross-lagged panel network in girls. Independent variables (i.e., predictors) are in columns, and dependent variables are in rows. Autoregressive edges are presented along the diagonal.

L1 = low fruit and vegetable consumption; L2 = sugar-sweetened-beverage consumption; L3 = breakfast skipping; L4 = outdoor activity time; L5 = TV screen time; L6 = computer time; L7 = mobile screen time; A1 = academic achievement; H1 = obesity; H2 = high blood pressure; H3 = high myopia; H4=depression.

Figure S1. Edge weight accuracy for the network of the total sample.

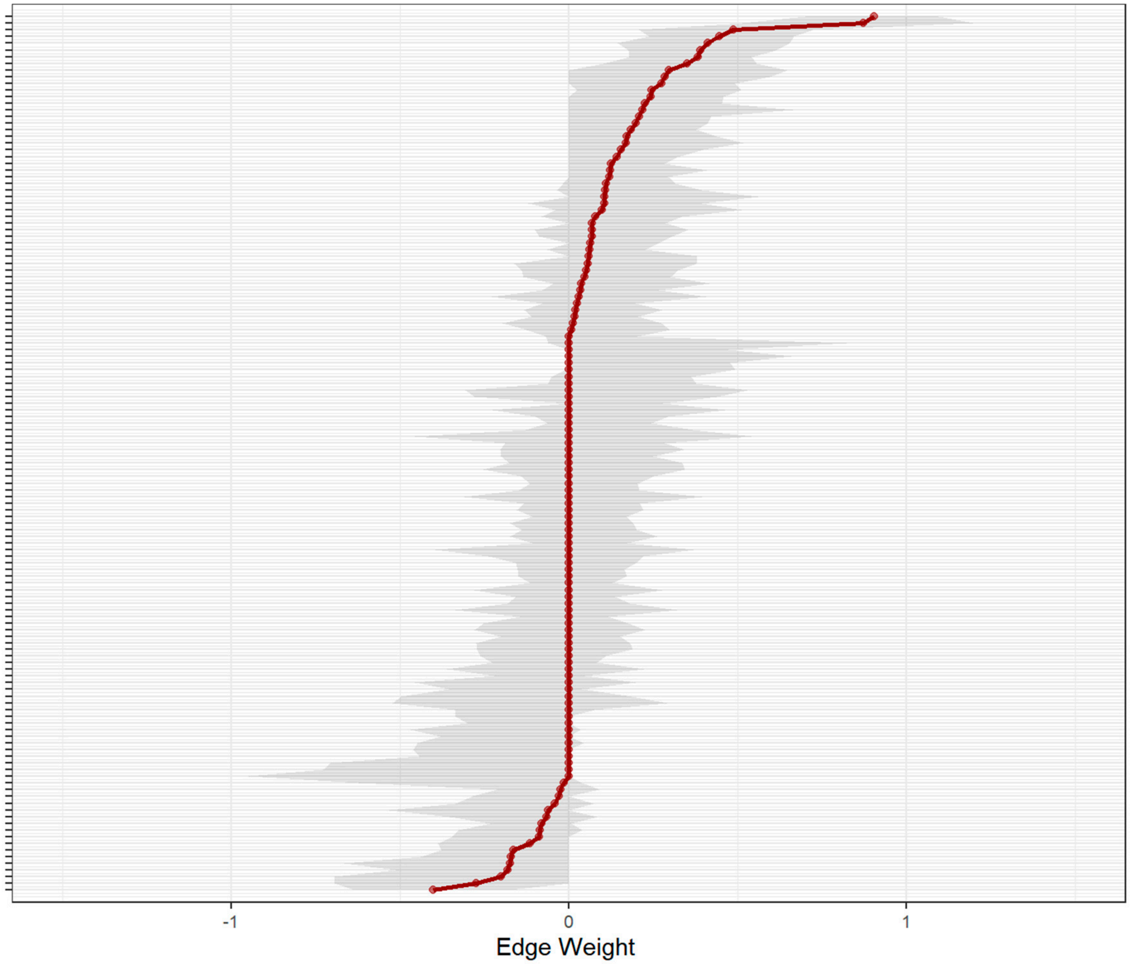

Figure S2. Edge weight accuracy for networks by sex.

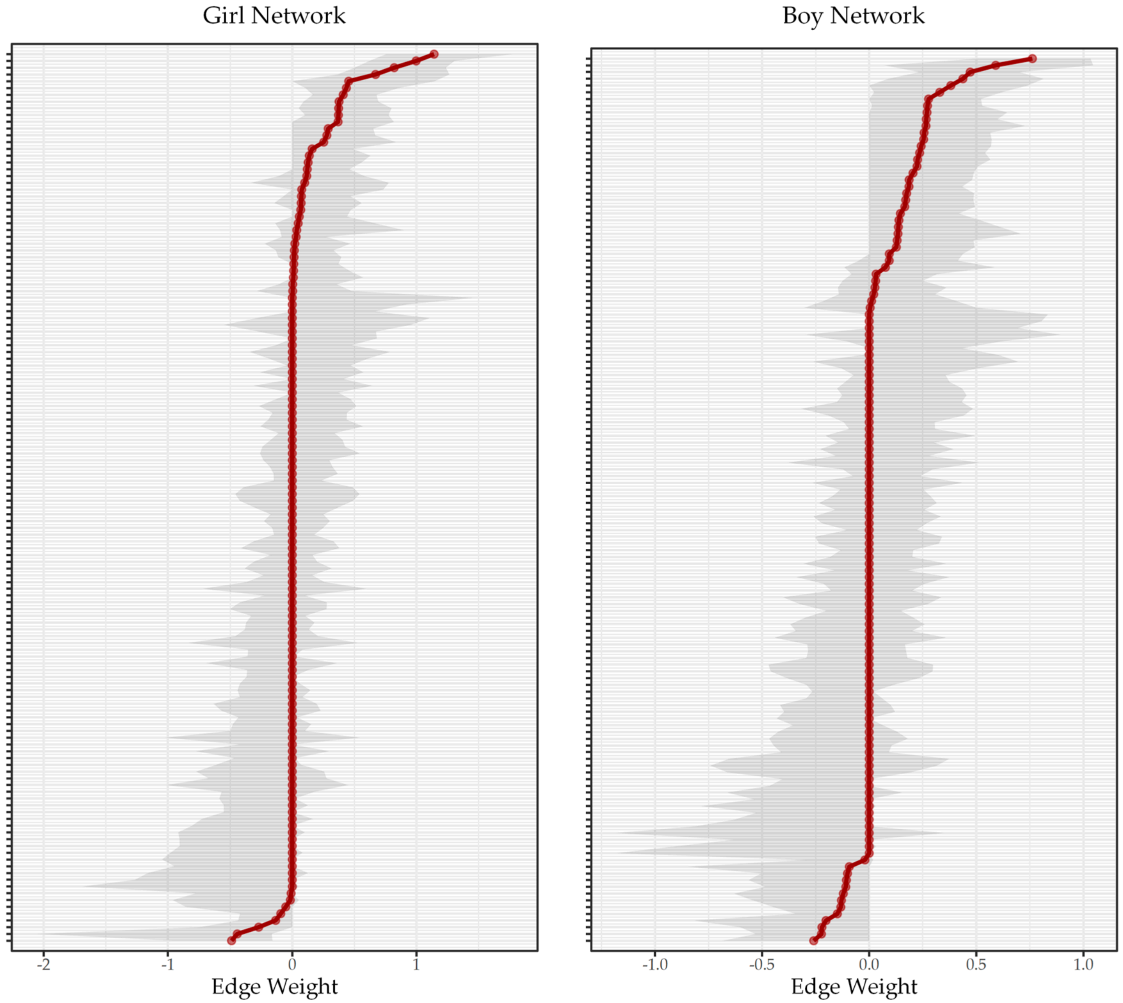

Figure S3. Stability of the centrality measures for the network of the total sample.

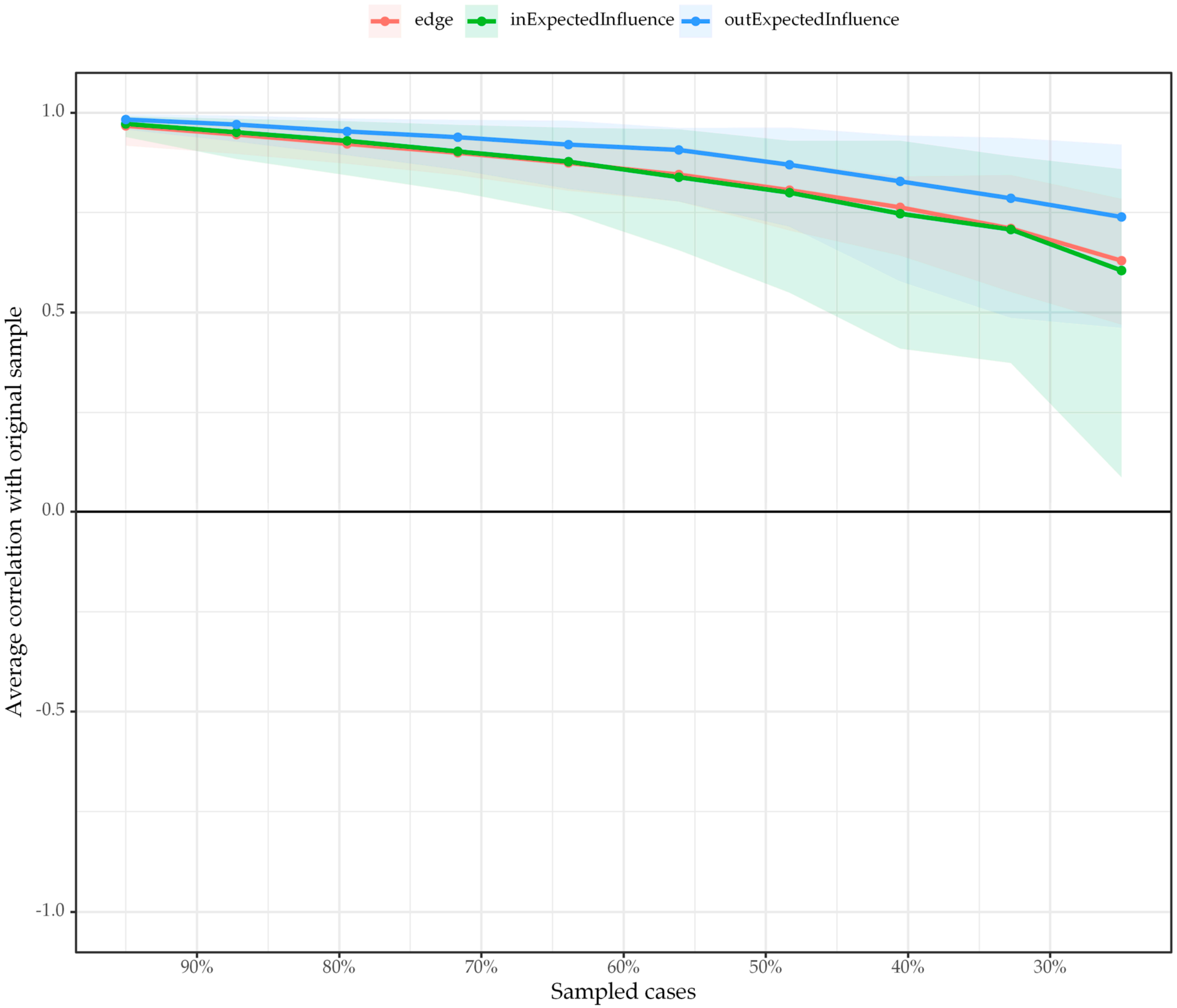

Figure S4. Stability of the centrality measures for networks by sex.

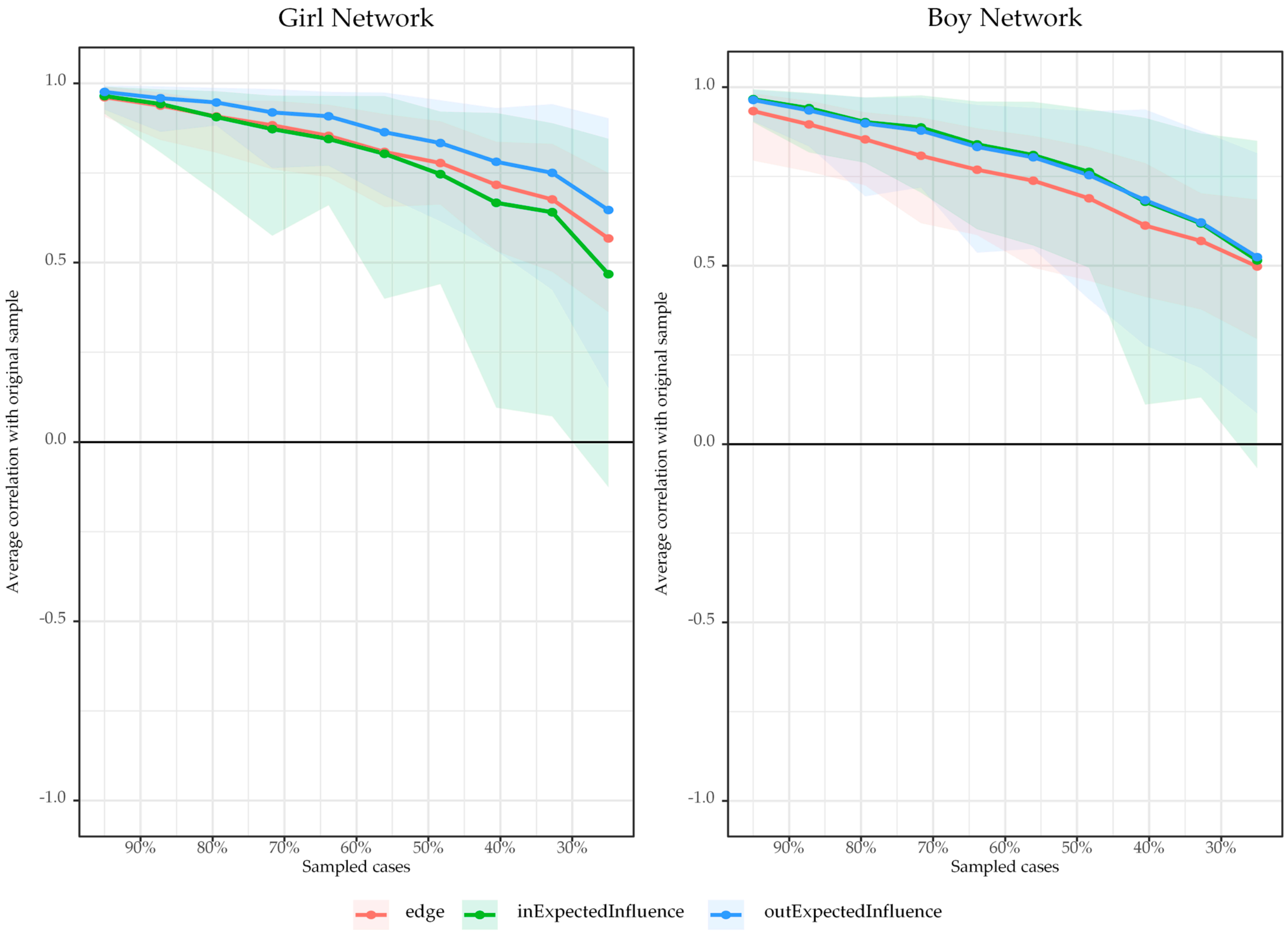

Figure S5. Edge weight difference tests in the network of the total sample.

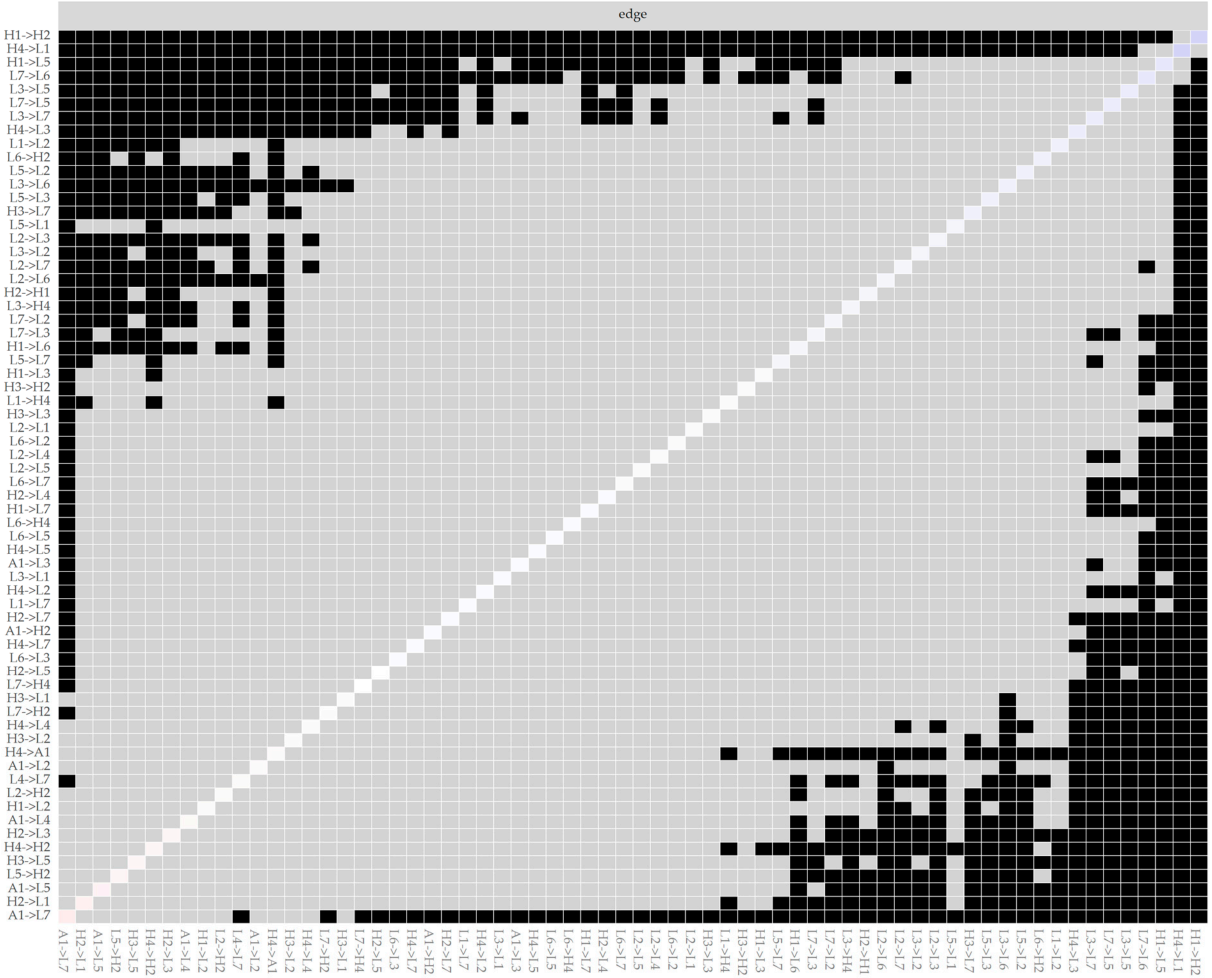

Figure S6. Edge weight difference tests for the networks by sex.

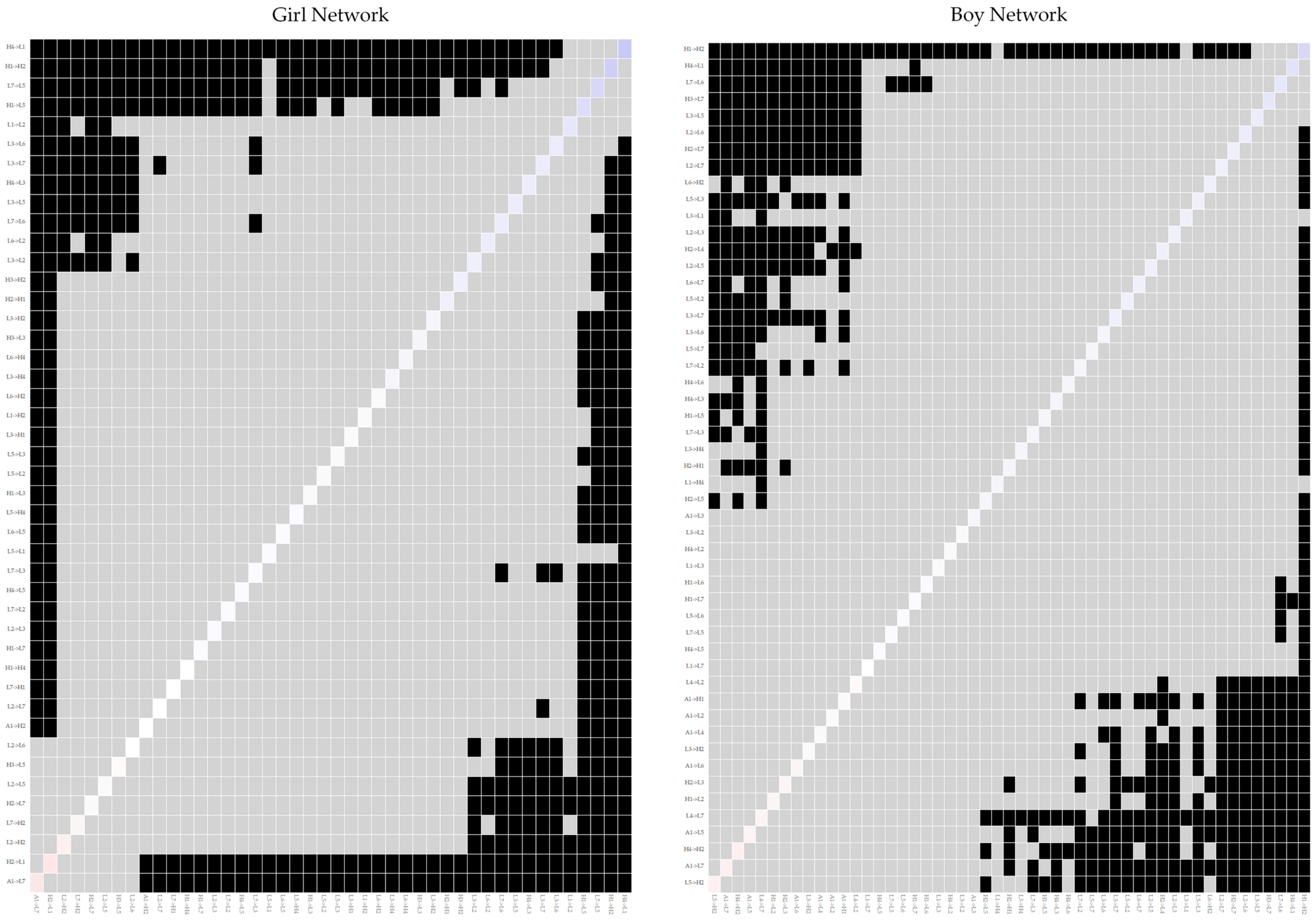

Figure S7. Out-EI and in-EI centrality difference tests in the total sample.

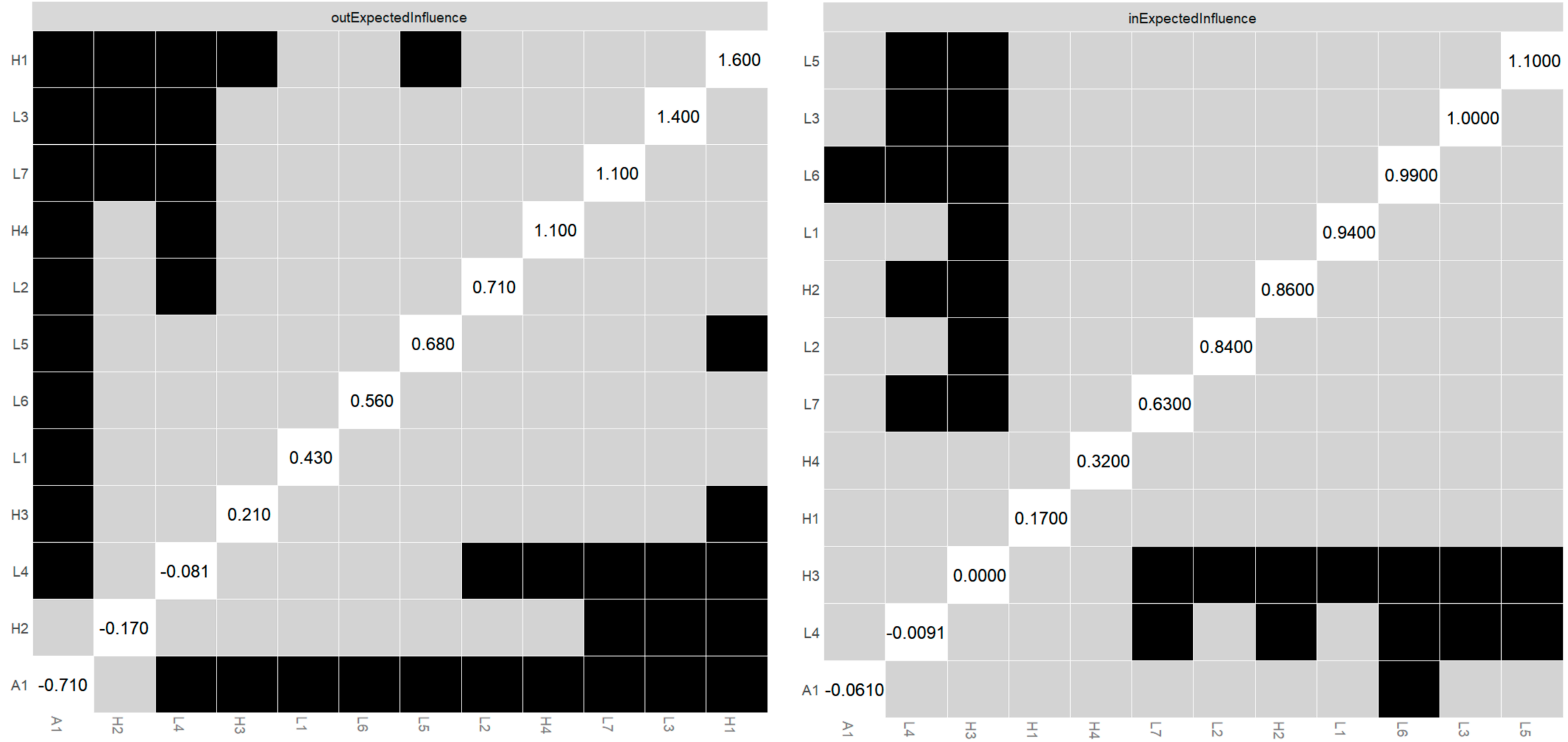

Figure S8. Out-EI and in-EI centrality difference tests in boys.

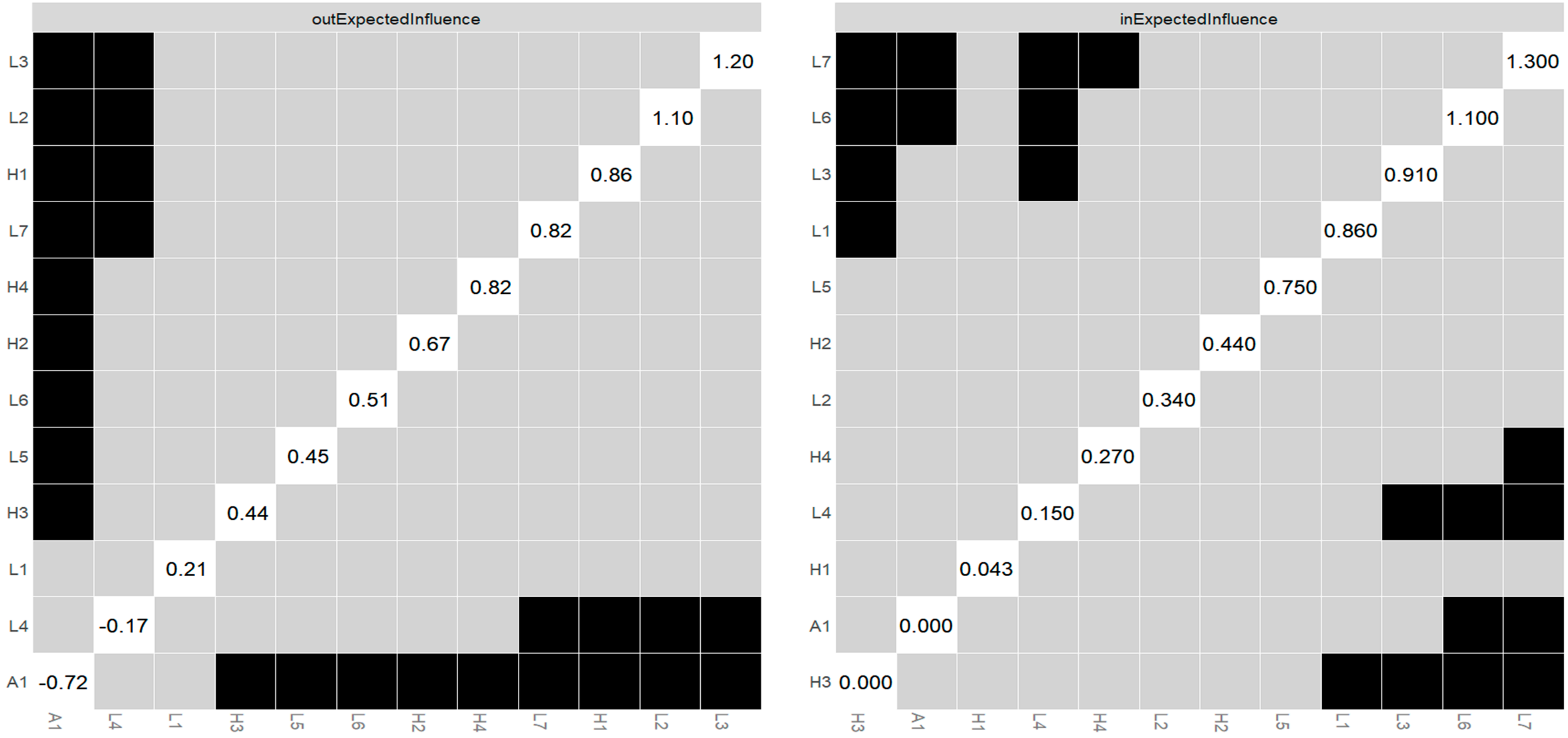

Figure S9. Out-EI and in-EI centrality difference tests in girls.

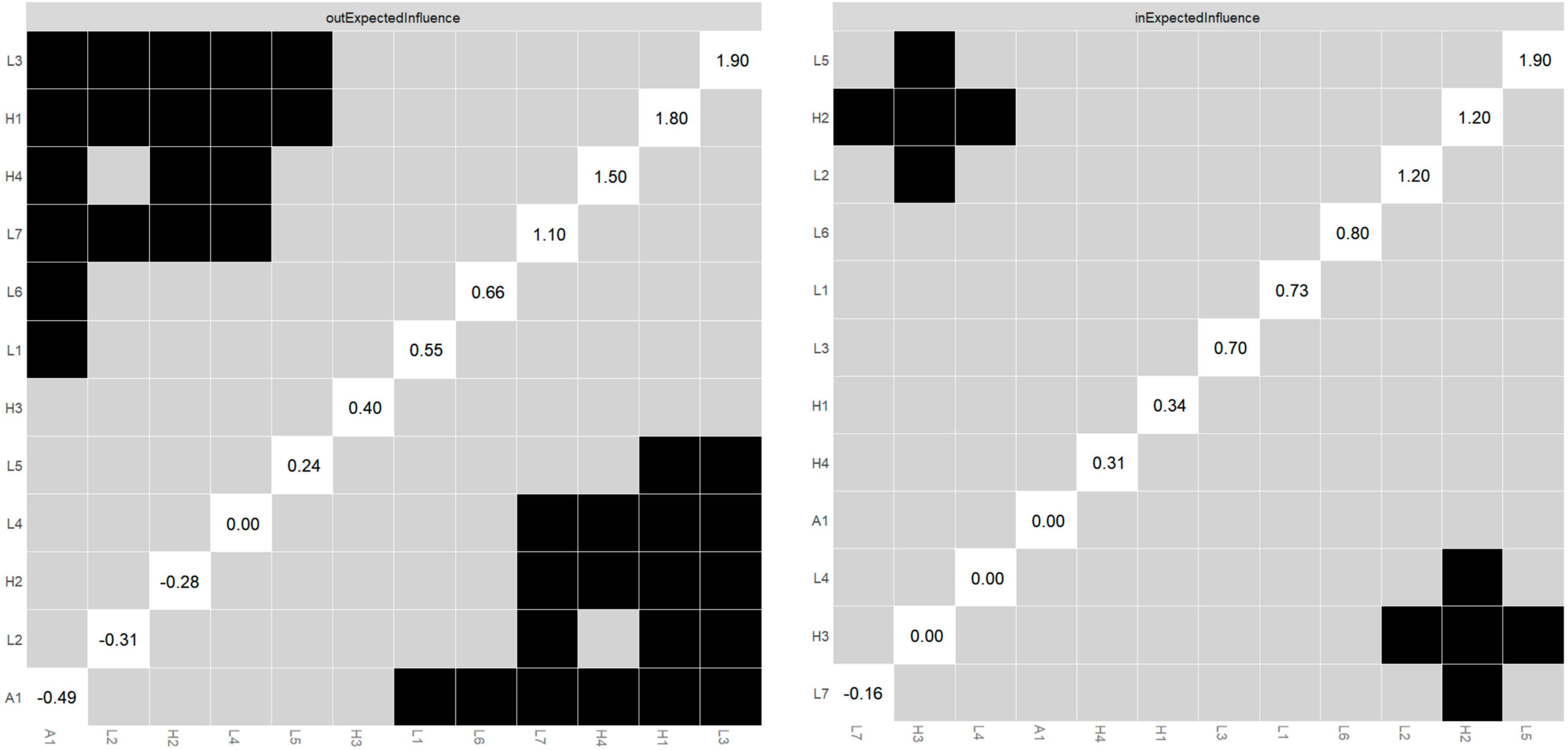

Supplement: Supplementary file 1 [file nutrients-18-00440-s001.zip › nutrients-4002731-supplementary.pdf]
